# Developing an Evidence-Based Nursing Handover Standard for a Multi-Site Public Hospital in Switzerland: Protocol for a Web-Based, Modified Delphi Study

**DOI:** 10.2196/15910

**Published:** 2020-01-08

**Authors:** Nadine Tacchini-Jacquier, Els de Waele, Peter Urben, Pierre Turini, Henk Verloo

**Affiliations:** 1 Development of Nursing Practices Unit Valais Hospital Sion Switzerland; 2 Haute École Spécialisée de Suisse Occidentale Valais Hospital Sion Switzerland; 3 School of Health Sciences Haute École Spécialisée de Suisse Occidentale Valais / Wallis University of Applied Sciences of Western Switzerland Sion Switzerland; 4 Service of Old Age Psychiatry, Cery Lausanne Switzerland

**Keywords:** modified e-Delphi survey, consensus, nursing, shift, nursing handover, standard, inpatient transfers, evidence-based practice, multi-site hospital

## Abstract

**Background:**

Poor communication processes create opportunities for errors when caregivers fail to transfer complete and consistent information. Inadequate or nonexistent clinical handovers or failures to transfer information, responsibility, and accountability can have dire consequences for hospitalized patients. Clinical handover is practiced every day, in a multitude of ways, in all health care settings.

**Objective:**

The goal of this study is to build a consensus, evidence-based nursing handover standard for inpatients during shift changes or internal transfers between hospital wards. The study will be based on papers published by Slade et al.

**Methods:**

This protocol describes a modified Delphi data-collection survey involving a targeted panel sample of 300 nurse experts. A multi-round survey will select an anonymous panel from a multi-site public hospital in Switzerland. Each survey stage will be described and will build on the previous one. The study will end with a focus group discussion involving a randomly selected panel to explain why items for the evidence-based clinical nursing handover standard were accepted or not accepted. An item must achieve a consensus of ≥70% for inclusion.

**Results:**

The present study’s expected outcome is a consensus-built, evidence-based nursing handover standard for inpatients during shift changes or internal transfers between the wards of a multi-site public hospital in Switzerland.

**Conclusions:**

This survey will enable us to develop an evidence-based nursing handover standard for use during shift changes and internal inpatient transfers in a multi-site public hospital in Switzerland.

**International Registered Report Identifier (IRRID):**

DERR1-10.2196/15910

## Introduction

### The Rationale for an Evidence-Based Nursing Handover Standard

The complexity of health care and its communication processes continues to challenge health care professionals, institutions, and organizations. Poor communication processes create opportunities for errors when caregivers fail to transfer complete and consistent information [1]. Clinical handover is practiced every day, in a multitude of ways, in all health care settings [2,3]. Clinical handover of patients relates to, and is defined as [4]:

The transfer of professional responsibility and accountability for some or all aspects of care for a patient, or group of patients, to another person or professional group on a temporary or permanent basis.

The literature identifies three basic components of good practice in nursing handover styles: bedside, verbal, and nonverbal. Handovers at bedside are located at the patient’s bedside, which promotes patient and nurse face-to-face interaction and encourages patients’ verbal participation, thus making the patient central to the information exchange process [5,6]. Verbal communication is in an office setting, where the nurse responsible for a group of patients exchanges relevant, documented information. Nonverbal communication is in an office setting, where nurses inform themselves by reading the patient’s health record, which includes progress notes, medication charts, observation charts, and nursing care plans. However, there is also taped communication, which is in an office setting where the nurse in charge collects the relevant information and records it onto an audiotape so that the oncoming shift can listen at a convenient time.

Inadequate or nonexistent handovers, or failures to transfer information, responsibility, and accountability can have direct consequences for patients [7]. They can result in delays to diagnosis, treatment, and care, tests being missed or duplicated, and subsequent incorrect operationalization of care plans or drug follow-up [8]. Current nursing handover practices in a multi-site hospital in Switzerland are highly variable, unreliable, and differ across medical specialties. This can lead to discrepancies in the content and accuracy of the information provided. Previous studies have revealed multiple barriers to communication within health care organizations, including hierarchy, gender, ethnic background, primary health care education, and differences in communication styles [9,10]. These inconsistencies in communication cause considerable risks to patient safety and care [11]. Health care institutions have recently sought to discover specific risks and contributing factors that cause difficulties in handover communications [12]. An internal survey of health care professionals in a multi-site public hospital in Switzerland concerning the culture of safety in patient care in 2017 revealed that almost two-thirds of the health care professionals (nurses, physicians, and allied health care professionals) considered the quality of information transmission to be poor and at-risk to patient safety [13]. Experimental studies have shown that information is poorly retained if verbal or handwritten handovers are transferred across multiple shifts [14].

Validated root causes for communication failures at handover include: institutional cultures which fail to promote successful handovers (eg, lack of teamwork and respect), differing expectations between information givers and receivers, ineffective communication methods (whether verbal, recorded, bedside, or written), poorly timed or nonsynchronous physical transfers and patient handovers, insufficient time allocated to successful handovers, interruptions to handovers, lack of standardized procedures for conducting successful handovers, inadequate staffing to accommodate successful handovers at certain times of the day or week, and a lack of patient participation during the handover [15-17].

The web-based, modified Delphi (e-Delphi) survey proposed in this study will target the development of standardized solutions to those risks, to be followed by the development and implementation of factors to improve the effectiveness of communication during transitions of care [18]. It has been suggested that standardizing the content and processes involved in patient handovers (eg, shift reports) ensures consistency in the exchange of critical information and is an effective means of improving communication and thus patient safety [19,20]. Although there is a lack of detail about what the specific content necessary for handover communication should be, standardizing processes (eg, presenting the patient) could be a starting point for selecting the content (eg, patient name, age, and current condition). Effectively addressing the challenges of transferring information in complex care environments requires that specific information on each topic be incorporated into two-way communication [21]. The literature reveals little empirical evidence of a link between effective information transfer during handovers and patient safety [22].

### Rationale for a Web-Based, Modified Delphi Study

Delphi studies are a recognized method for building consensus around an issue where little knowledge or agreement previously existed [23]. The Delphi method is a framework for a forecasting process based on the results of multiple rounds of questionnaires sent to a panel of experts. This approach uses [24,25]:

Structured anonymous communication between experts to gather consensus perspectives about an issue or topics that can then be used to inform decision-making or to agree about methods of functioning.

Inadequate communication during nursing handovers increases the risk of adverse events because incomplete, inaccurate, or omitted data create ambiguities between the sender and the receiving health care professionals.

The web-based, modified Delphi study involves rounds of web-based questionnaires for which experts are asked to provide their opinions on particular topics [26]. Initially, this is done independently, but in subsequent rounds, the experts are made aware of the group’s opinions when making their decisions, to reach consensus. The key features of e-Delphi methods are that they are iterative and anonymous, which is particularly beneficial for a multi-site hospital with different medical specialties. Anonymity and the web-based format encourage participation and opinion-sharing by a large set of panel members, and they thus prevent dominant individuals from controlling discussions, which is important within hierarchical environments such as health care institutions. We will use a Delphi panel approach to find a consensus on data gaps and the most appropriate study design for constructing a standard evidence-based tool.

Higher numbers of handovers pose greater risks to patients, although little is known regarding the precise mechanisms by which handovers undermine care. Some studies have highlighted information management at shift changes as being particularly vulnerable to error [14,22,25,27]. Handover strategies should recognize the interconnectedness of the different categories and the themes of administrative, clinical, and medical information from the outset. The general themes of clinical nursing handover standards cover a range of factors which come together to define the degree of smoothness and patient safety involved in those handovers [28-30]. Handovers are an essential component in the continuity of care [31]. During the patient journey, such transitions in care are notably vulnerable periods [32]. Transferring the responsibility of care to another practitioner introduces the potential for an error to occur should all the relevant information not be communicated accurately and efficiently [33]. The information transferred may be inaccurate, lack clarity, or be incomplete, which increases the risk of potentially harmful errors [34,35].

### Study Purpose

The purpose of this study is to outline a proposed protocol for an e-Delphi survey to construct a consensus, evidence-based nursing handover standard for inpatients during shift changes or internal patient transfers between the wards of a multi-site public hospital in Switzerland. The study has not yet been conducted.

## Methods

### Design

This protocol describes a multi-round survey of an anonymized panel selected from a multi-site public hospital in Switzerland to find a consensus for an overall, evidence-based nursing handover standard for nursing shift changes and internal inpatient transfers. In the absence of a standardized reporting protocol for a modified e-Delphi survey, this protocol referred to publications by Keeney et al [36], Burchell et al [37], Slade et al [38,39], and Cole et al [23].

### Setting and Population

The study will be conducted in a multi-site public hospital, located in a southern part of Switzerland, and serving a population of about 340,000 [13]. It recorded almost 40,000 individual hospitalizations in 2017 and is composed of two hospital centers in two linguistically and culturally different regions [13]. Each hospital center includes standard medical hospitalization wards to cover its mission of universal public health care.

The research sample population will be composed of a panel of 300 eligible nurse experts drawn from the institution’s staff lists of clinical nurse specialists and nurse supervisors. They are all highly qualified, experienced, and recognized within their departments. In agreement with the two hospital centers’ directors, all eligible nurse experts will be invited to participate in the e-Delphi survey.

### Eligibility and Recruitment of the Web-Based, Modified Delphi Panel of Nurse Experts

In our application of the e-Delphi method, the term expert includes people with significant work experience as health care professionals. The investigators will invite professionally active nurse experts from the domains of general medicine, surgery, geriatrics, rehabilitation, gynecology, obstetrics, pediatrics, emergency department medicine, intensive care, anesthesia, and psychiatry to join the panel. The inclusion criteria will be: (1) to have worked in their current specialty for at least three months before the launch of the data collection process; (2) to have been employed as a registered nurse clinical educator, a student-success coach, or a nurse supervisor; and (3) the capacity, willingness, and time to participate in the expert panel and the communication skills to understand and answer clinical statements effectively.

### Literature Review to Design the Components of the Web-Based, Modified Delphi Survey

**Figure 1:**
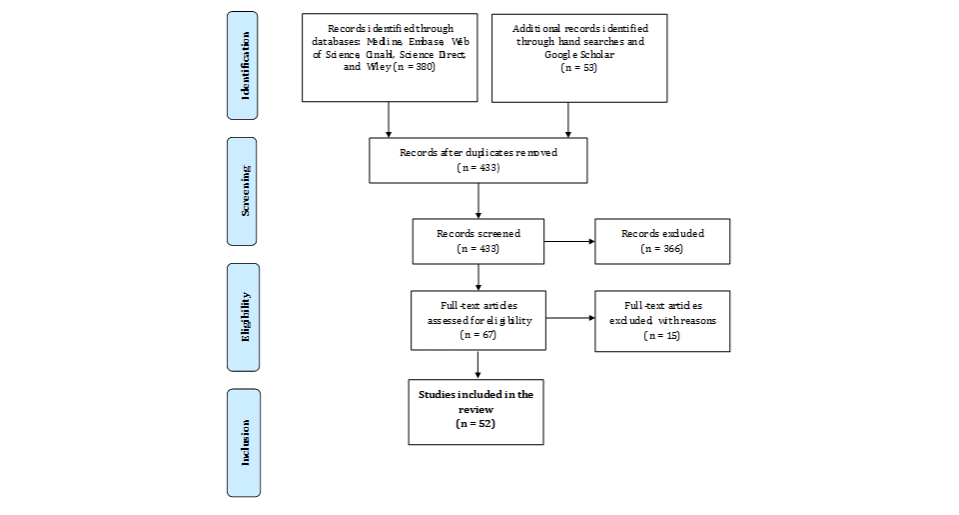
Search strategy for the retrieval of the necessary components of a nursing handover standard.

A literature review was conducted to map the components needed for an evidence-based, clinical nursing handover standard. A comprehensive scoping review of all the components of effective, evidence-based nursing handovers has been conducted, as have research group discussions on the necessary content for those handovers (see Figure 1 and Textbox 1) [40]. In collaboration with a medical librarian and using predefined search terms, we conducted a comprehensive scoping literature search for published articles in the following electronic databases, from inception until September 30, 2018: Medline via PubMed (from 1946), Embase (from 1947), CINAHL (from 1937), the Web of Science (from 1900), Science Direct, and Wiley. We conducted a hand search of the bibliographies of all relevant articles and completed this with a search for unpublished studies using Google Scholar.

Inclusion criteria for our literature search were that the article described the definition, content (structure), and implementation of a nursing handover standard. The search accepted study designs that were either uncontrolled trials, trials with a comparison group (uncontrolled, pre-post historical controls), or observational studies. Publications could be in English, French, or German, but they were excluded if they focused on interprofessional handovers, transitions from hospital to home, primary care settings, or they were study protocols. The search syntax consisted of the search themes joined using the Boolean terms “AND” and “OR”. The following descriptor terms and free keywords were included: “shift-to-shift hand-off,” “hand-off,” “handover,” “sign-out,” “shift change,” “shift change report,” “inter-shift report,” “shift-to-shift report,” “transition of care,” AND “nursing; nursing care; nursing quality; nursing safety; inpatients; hospital; hospitalization.” Figure 1 presents the search strategy’s results.

**Relevant components of an evidence-based nursing handover standard for inclusion in the e-Delphi survey.**

**Culture and attitude for good handover practices:**

- Respectful and collaborative attitude
- Proactive listening
- Positive, factual language adapted to patients, situations, and professionals
- Confidentiality
- The handover environment

**Handover preparation, including coordination and sources of information:**

- Clinical assessment before the handover
- Use different sources of information
- Updated patient records
- Reconsider and reanalyze information

**Handover phases, including communication of patient-specific information**

- Mnemonic techniques to guide communication and format content chronologically
- Face-to-face handovers with the opportunity to ask questions
- Information technology to support data access to the patient’s complete history and health status
- Patient records ensuring the traceability of decisions and follow-up
- Information technology to support data updates
- Flexible information technology to support adaptations for each specialized ward
- Handovers at the patient’s bedside at the risk of reduced confidentiality
- Handovers at the patient’s bedside for understanding their values and preferences

**A minimum dataset should be transmitted:**

- Summary of the patient’s hospitalization history and care planning
- Assessment of the disease
- Prognosis of health status
- Allergies
- Reanimation status
- Medication treatment
- Laboratory results
- Vital signs
- Patient’s activities and planned examinations

### Knowledge Synthesis for the Selection of Items for the Nursing Handover Standard

The investigators reviewed the above findings at two meetings and selected the relevant components of an evidence-based nursing handover standard to be included in the Delphi panel survey. Textbox 1 presents the main components of an evidence-based nursing handover standard retrieved in the comprehensive scoping review.

### Web-Based, Modified Delphi Survey Administration

Each round of the e-Delphi survey will be communicated through SurveyMonkey, which is a secure, commercial, web-based platform that ensures anonymous survey participation. The collected data will be stored in Switzerland, protected with high-end firewalls, and treated confidentially. All the eligible nurse experts and potential members of the expert panel will be sent a personalized link to fill out each round of the survey. Although a personalized link is used to access the survey, personal information will not be stored, and contact details will be removed from the completed survey. The survey’s predetermined percentage of agreement to qualify as consensus was established at 70% for all the items in the e-Delphi process’ different rounds [36].

### Rounds in the Web-Based, Modified Delphi Process

The e-Delphi data collection process will be composed of three rounds. Item management and answers from each round will be downloaded into the SPSS 25.0 software package for analysis (IBM Corp, Armonk, New York, United States). These enabled the development of a structured questionnaire linking all the possible components with item statements on which should be included in the handover standard. Participants will give their opinions on whether items should be included by using a five-point Likert scale ranging from strongly agree to strongly disagree. One open-ended question at the end of the questionnaire will ask:

What topic, not yet mentioned in these statements, should also be integrated into the handover standard?

The questionnaire will be translated into French and German and trialed with three or four clinical experts not involved in this e-Delphi survey.

In the first round, the potential panel of experts will be asked to respond to a selection of items (Textbox 1). This will involve distributing the structured questionnaire by email to the selected sample of potential participants, including a cover letter describing the study’s aim and instructions on how to fill in the questionnaire. Respondents will be able to use an open text field (with the open-ended question) to explain their choices or suggest items not listed in the first round, but which they believe are important. Finally, the panel will also report their sociodemographic and professional characteristics, including age, professional role, and years of experience. An email reminder will be sent out every week after launching the e-Delphi process. The first round will close after 30 days, and the returned data will be analyzed.

The second round will only involve those statements for which no consensus agreement was found in the first round, plus new statements which have arisen from the panel’s suggestions in response to the open question. A cover letter explaining this, and with further instructions for round two, will be sent with the questionnaire. A weekly reminder will be sent out. The second round will end after 30 days, and the returned data will then be analyzed. The second round’s expected outcome will be a final selection of items about which the panel agreed. Moreover, if the items generated by the open question did not find any consensus in the second round, they will be resubmitted in the third round.

The third and final round will end with a focus group cognitive debriefing made up of 8-10 randomly selected, but highly involved, nursing experts. Cognitive debriefing is the process by which the results of a survey are actively discussed among representatives of the target population [41].

**Figure 2:**
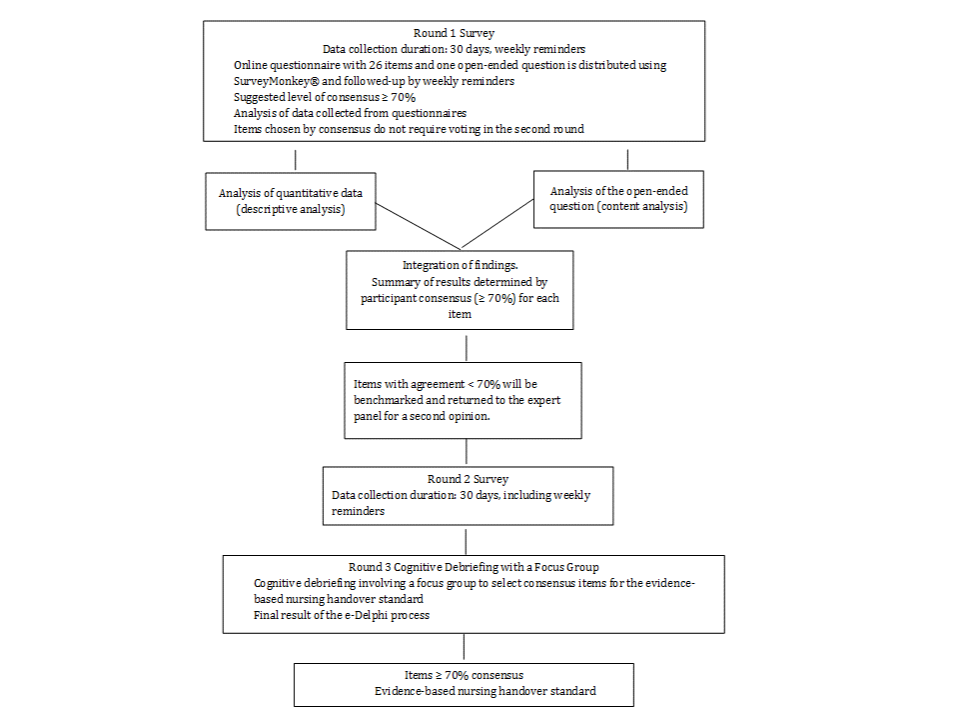
Planned e-Delphi survey data-collection process to design an evidence-based nursing handover standard. e-Delphi: web-based, modified Delphi.

Numerous qualitative methods have been used to capture caregivers and clinicians’ perspectives of important changes in daily practice. Patrick et al considered that the cognitive debriefing technique could be used in a Delphi study [42]. As Patrick et al outlined, the cognitive debriefing process is structured around and usually focused upon the assessment of a specific clinical output, and it incorporates direct questions about the understandings, relevance, and comprehensiveness of the measures leading to that output [42]. They will explain and validate the consensus items to be used in the standard for evidence-based nursing shift handovers and patient transfers within our multi-site public hospital in Switzerland (Figure 2). The focus group guide will be pretested before the start of the focus group.

### Data Analysis

The analysis of responses from each round will be done iteratively and independently. The investigators will supplement their quantitative analyses with a thematic analysis of the text of the open-ended question to better understand disagreements and include suggestions [38].

The proposed study will be carried out using the SurveyMonkey online questionnaire website, and all the items will be translated into French and German. Data will be extracted into an Excel (Microsoft, Redmond, Washington, United States) spreadsheet and then imported into SPSS statistical software, version 25. The population of panel experts will be described using descriptive statistics such as frequencies, distributions, and leading trends. The data collected on a 5-point Likert scale will be recoded as dichotomous variables: disagreeing with the statement will include the strongly disagree, partially agree, and no opinion responses, and agreeing with the statement will include the partially agree and strongly agree responses. Consensus agreement on inclusion by the nurse experts versus no consensus on inclusion will be calculated by using the sum of the distribution of the agree/disagree responses for the item. As there is no standard definition of consensus for Delphi studies [43], we have chosen a consensus level of ≥70% of responses selecting the item for inclusion in the handover standard [36]. Items that do not reach the required level of consensus in round one will be reconsidered in round two. Appropriate exact tests will be used to compare means and percentages if participant anonymity can be ensured. Analyses for internal validity will be carried out by three or four experienced nurse experts who were not included in the proposed study and who will be contacted by email and asked to examine the questionnaire items’ clarity, wording, and understandability before the modified e-Delphi study is launched.

### Ethical Considerations

Ethical approval has been obtained from the Human Research Ethics Committee of the Canton Vaud (CER-VD) (2019-00925). The study will ensure the anonymity of its panel of nurse expert participants as well as the standards of good research practice mentioned in the Declaration of Helsinki [44].

## Results

The present study’s expected outcome is a consensus-built, evidence-based nursing handover standard for inpatients during shift changes or internal transfers between the wards of a multi-site public hospital in Switzerland. The first round’s expected outcomes will be the selection of numerous items about which the panel agreed and a list of items about which it could not find a consensus. The second round’s expected outcomes will be a final selection of the items about which the panel was in agreement and a list of items about which no agreement could be found (ie, topics rejected for inclusion in the handover standard). The third round of the e-Delphi process will be a cognitive debriefing involving a focus group discussing the consensus/no consensus items in the nursing shift handover and patient transfer standard.

## Discussion

### Primary Findings

The significant number of nurse experts involved in the proposed e-Delphi survey determined our use of an electronic data collection method. However, different data collection methods for Delphi surveys, such as using face-to-face interviews or focus groups with and without patients, have been previously documented in the literature [36,45,46]. The study’s potential for introducing significant benefits to the patient handover and transfer contexts lies in its combined use of clinical and applied research skills to meet a patient safety issue. Indeed, the study will have a direct impact on future patient safety, quality of care, and the continuum of care. It will enable the front-line nursing staff of a multi-site public hospital in Switzerland to coconstruct their consensus on the content necessary for an internal, evidence-based nursing shift handover and patient transfer standard. In addition to the items to be included in the nursing shift handover standard, the proposed study will seek a consensus about information flows and patients’ involvement in their nursing handovers. The proposed study’s findings will be a substantial contribution to the hospital’s overall strategy for continually improving the quality and safety of care using evidence-based practices [47]. The investigators’ choice of the e-Delphi method maintains the principles of offering an equal voice to all professional stakeholders. As Klee et al and McFarlane mentioned, a consensus handover standard is a way to change the daily practice of all the nurses involved in a hospital’s nursing processes, and it is not uniquely limited to those survey participants who accepted the items for the standard [48,49].

The proposed study design is not without potential pitfalls. One significant challenge was the selection of the items for the nursing handover standard. The investigators’ choices were based on a comprehensive scoping review. Nevertheless, it is possible that specific important topics, which may be effective in our settings and context, were overlooked or excluded. A second challenge will be ensuring a representative selection of nurse experts, considering the particularly heterogeneous characteristics of their training and clinical expertise (management versus clinical experts). The investigators will seek to make the sample as representative by identifying diverse perspectives, but it should be acknowledged that the opinions of the nurse experts selected are unlikely to capture every possible viewpoint. Involved nurse experts will ensure that item rating is carefully considered, and the response rates remain high. Input will be maximized by limiting the number of questions asked in each round. The focus group will enable a better understanding of why nurse experts did or did not select specific items by giving them a voice and assessing their experience of an e-Delphi study. Procedures will be established to minimize participation by ineligible staff, including the active promotion of the study within the hospital and direct invitations to certain potential participants, via established, official staff lists.

Finally, certain items may not reach the desired level of consensus, even after three rounds. However, by conducting the e-Delphi survey, the investigators will have developed a better understanding of the importance of accurately and effectively transmitting relevant information between nurses during shift changes and internal patient transfers, and they will be better prepared and positioned to anticipate any potential barriers to implementation.

Future additional research will be considered, such as an implementation study of the handover standard tool in the different acute wards, a before-after study after the introduction of the evidence-based nursing handover tool, and a qualitative study of perceptions of nurses and patients about the introduction of a standard handover tool in daily practice.

### Conclusion

The results of the proposed e-Delphi study will characterize the perceived requirements for an evidence-based nursing handover standard. However, the methodology of this e-Delphi survey in a multi-site public hospital in Switzerland should not be considered generalizable for building clinical nursing handover standards for other hospitals. Nevertheless, the approach presented here could be thought of as good clinical research practice for surveying a large sample and may be transferable to other settings where the input of experts in a particular field is required to coconstruct methods of safely transmitting information between health care professionals.

## Abbreviations

e-Delphi: web-based, modified Delphi
